# Potential Protective Effect of Osteocalcin in Middle-Aged Men with Erectile Dysfunction: Evidence from the *FAMHES* Project

**DOI:** 10.1038/s41598-018-25011-9

**Published:** 2018-04-30

**Authors:** Yang Chen, Jie Li, Jinling Liao, Yanling Hu, Haiying Zhang, Xiaobo Yang, Qiuyan Wang, Zengnan Mo, Jiwen Cheng

**Affiliations:** 1 Institute of Urology and Nephrology, The First Affiliated Hospital of Guangxi Medical University, Nanning, China; 2 Center for Genomic and Personalized Medicine, Guangxi Medical University, Nanning, Guangxi Zhuang Autonomous Region China; 3 The Guangxi Zhuang Autonomous Region Family Planning Research Center, Nanning, Guangxi China; 4 Department of Urology, The First Affiliated Hospital of Guangxi Medical University, Nanning, China; 5 Guangxi collaborative innovation center for genomic and personalized medicine, Nanning, Guangxi Zhuang Autonomous Region China; 6 Guangxi key laboratory for genomic and personalized medicine, Guangxi key laboratory of colleges and universities, Nanning, Guangxi Zhuang Autonomous Region China

## Abstract

In a similar manner to erectile dysfunction (ED), osteocalcin (OC) is also said to be associated with cardiovascular disease (CVD); however, the effect of OC in ED is unclear. This study was conducted based on the Fangchenggang Area Male Health and Examination Survey (*FAMHES*) project that ran between September and December 2009. ED was evaluated using the International Index of Erectile Function (IIEF-5). OC was shown to be associated with mild (unadjusted: OR = 0.647; *P* = 0.016) or moderate (unadjusted: OR = 0.453; *P* = 0.007) ED. Meanwhile, higher OC levels were more prominently associated with ED (unadjusted: OR = 0.702; *P* = 0.014). When subdividing the groups by age, the correlation between OC and ED presented in those aged 40–49 years, even in the multi-adjusted model, for those with moderate (OR = 0.255, *P* = 0.044) and severe (OR = 0.065, *P* = 0.005) ED. The relationship between OC and ED was also associated with a high level of testosterone, non-obesity, drinking, and non-metabolic syndrome. In summary, OC may play a protective role in middle-aged (40–49 years) men with moderate-severe ED, especially those with a high level of testosterone, non-obesity, drinking, and non-metabolic syndrome.

## Introduction

Osteocalcin (OC) is a non-collagenous protein derived from osteoblasts^1^, which can be γ-carboxylated (Gla) at one or more glutamic acid (Glu) residues^2,3^. It was first identified as a marker of bone formation^4^. In 1996, OC-deficient mice (Osn^−/−^) were shown to have increased visceral fat and insulin resistance^5^. This phenomenon was explained in 2007 when OC was shown to affect energy metabolism by favoring β-cell proliferation, insulin secretion, and insulin sensitivity^6^. In 2011, the same research group also showed that OC could influence the reproductive system and testosterone level^7^. In addition, it was recently suggested that OC could be associated with cardiovascular disease (CVD) and reduce the risk of CVD in older men^8^. Moreover, in 2016, Ling *et al*.^9^ identified a potential correlation between OC gene polymorphisms and blood pressure.

As one of the common male urogenital diseases, erectile dysfunction (ED) is defined as the inability to acquire and maintain a sufficient erection for satisfying sexual intercourse. Among its various etiologies, the hormone and vascular components are dominant^10,11^. Moreover, one study also reported that ED may be a marker of CVD^12^; however, the association between OC and ED has not been discussed until now. This study was conducted to remedy this gap in the literature using data from the Fangchenggang Area Male Health and Examination Survey (*FAMHES*) project.

## Results

### The essential characteristics of subjects with and without ED

A total of 4303 participants (aged 18–88 years) were recruited to the *FAMHES* project between September and December 2009, of whom 3593 responded to further interviews. Rigorous criteria were applied to investigate the association between OC and ED, and a total of 1567 eligible samples were selected for the analysis. The IIEF-5 system defined two groups: 746 individuals with ED (IIEF-5 ≤ 21) and 821 individuals without ED (IIEF-5 > 21). The baseline results suggested that those with ED were significantly older than those without ED (ED vs. non-ED: 38.14 ± 1.82 vs. 33.99 ± 8.10 years; *P* < 0.001). In addition, abdominal obesity was also more serious in the ED patients (ED vs. non-ED: 0.89 ± 0.06 vs. 0.88 ± 0.06; *P*_WHR_ = 0.012; *P*_%_ = 0.044). Interestingly, the level of OC was lower in those with ED than those without ED (ED vs. non-ED: 24.38 ± 8.21 vs. 25.56 ± 8.64 ng/ml; *P* = 0.003) (Table 1).

**Table 1:** The baseline analysis for the eligible participants in this study.

	ED ≤ 21	No-ED > 21	P
NO.	746	821	
Age, years	**38.14 ± 1.82**	**33.99 ± 8.10**	**<0.001**
Osteocalcin, ng/ml	**24.38 ± 8.21**	**25.56 ± 8.64**	**0.003**
Testosterone, ng/ml	6.34 ± 1.85	6.23 ± 1.93	0.233
BMI, kg/m^2^	23.42 ± 3.26	23.30 ± 3.45	0.488
<24	436 (58.45%)	500 (60.90%)	
<28, ≥24	249 (33.38%)	244 (29.72%)	
≥28	61 (8.17%)	77 (9.38%)	0.259
WHR, %	**0.89 ± 0.06**	**0.88 ± 0.06**	**0.012**
≤0.9	**454 (60.86%)**	**540 (65.77%)**	
>0.9	**292 (39.14%)**	**281 (34.23%)**	**0.044**
Smoking, %			
Yes	410 (54.96%)	455 (55.42%)	
No	336 (45.04%)	366 (44.58%)	0.855
Drinking, %			
Yes	636 (85.25%)	726 (88.43%)	
No	110 (14.75%)	95 (11.57%)	0.063
MetS, %			
Yes	65 (8.71%)	68 (8.28%)	
No	681 (91.29%)	753 (91.72%)	0.760
MetS score, %			
0	292 (39.14%)	347 (42.27%)	0.217
1	232 (31.10%)	239 (29.11%)	0.341
2	131 (17.56%)	142 (17.30%)	0.894
3	66 (8.85%)	65 (7.92%)	0.523
≥4	25 (3.35%)	28 (3.40%)	1.000

*The ED is defined with the threshold of IIEF-5 of 22 score.

*Quantitative traits represented as mean ± SD are tested with student’s t test. Qualitative traits are tested with chi-square test.

*ED: erectile dysfunction; BMI: body mass index; WHR: waist hip rate; SD: standard deviation.

### Potential protective function of OC in ED

In the next analysis, regression analyses, including linear and binary regressions, were applied. Although no significant association between OC and ED risk was identified in the linear regression analysis, OC was significantly associated with ED risk in the binary logistic regression analysis (unadjusted: OR = 0.618, 95% CI = 0.449–0.850, *P* = 0.003) (Table 2). To further confirm this association, ED was divided into four groups: mild, moderate, severe, or no dysfunction. The multinomial logistic regression analysis was conducted using the no dysfunction group as the reference. The results suggested that OC may be predominantly related to the mild (IIEF = 17–21; unadjusted: OR = 0.647, 95% CI = 0.454–0.921, *P* = 0.016) and moderate (IIEF = 12–16; unadjusted: OR = 0.453, 95% CI = 0.255–0.804, *P* = 0.007) forms of ED (Table 3). Following these analyses, the OC level was divided into quartiles (Q1 < 25%, 25% ≤ Q2 ≤ 50%, 50% < Q3 ≤ 75%, Q4 > 75%). The results of the multinomial logistic regression analysis suggested that higher OC levels were more prominently associated with ED (Q4, unadjusted: OR = 0.702, 95% CI = 0.530–0.930, *P* = 0.014) (Table 4).

**Table 2:** The linear and binary logistic regression analyses for the association between osteocalcin, testosterone and ED.

	Unadjusted			Age-adjusted			Multi-adjusted		
	BETA/OR	95%CI	P	BETA/OR	95%CI	P	BETA/OR	95%CI	P
IIEF-5 score									
osteocalcin	0.566	−0.105, 1.237	0.098	−0.330	−1.030, 0.371	0.356	−0.131	−0.856, 0.593	0.722
testosterone	−0.625	−1.313, 0.063	0.075	**−1.004**	**−1.686, −0.323**	**0.004**	**−0.815**	**−1.591, −0.038**	**0.040**
ED									
osteocalcin	**0.618**	**0.449–0.850**	**0.003**	0.979	0.693–1.383	0.905	0.936	0.655–1.339	0.718
testosterone	1.272	0.919–1.762	0.147	**1.596**	**1.138–2.240**	**0.007**	**1.648**	**1.121–2.424**	**0.011**

*In the linear regression analysis, as a continuous variable the IIEF-5 score is treated as the dependent factor. In the binary logistic regression analysis, ED (dichotomous variable: ED or without ED) is treated as the dependent factor.

*ED: erectile dysfunction; BMI: body mass index; WHR: waist hip rate; OR: odd ratio; 95%CI: 95% confidence interval.

*Multi-adjusted: age, BMI, WHR, smoke and drink.

**Table 3:** The multinomial logistic regression analysis for the association between ED and osteocalcin.

	Unadjusted			Age-adjusted			Multi-adjusted		
	OR	95%CI	P	OR	95%CI	P	OR	95%CI	P
ED									
None (22–25)	1	1	1	1	1	1	1	1	1
Mild (17–21)	**0.647**	**0.454–0.921**	**0.016**	0.935	0.641–1.365	0.728	0.919	0.621–1.358	0.671
Moderate (12–16)	**0.453**	**0.255–0.804**	**0.007**	0.930	0.503–1.721	0.818	0.871	0.461–1.647	0.672
Severe (5–11)	0.795	0.379–1.665	0.543	1.359	0.621–2.970	0.443	1.133	0.502–2.557	0.764

^*^ED is divided into four groups according to the IIEF-5 scores: None = 22–25 scores, Mild = 17–21 scores, Moderate = 12–16 scores and Severe = 5–11 scores. None ED is treated as the reference.

^*^ED: erectile dysfunction; BMI: body mass index; WHR: waist hip rate; OR: odd ratio; 95%CI: 95% confidence interval.

**Table 4:** Results of multinomial logistic regression analysis for the association between ED risk and osteocalcin level.

	Q1	Q2			Q3			Q4		
		OR	95%CI	P	OR	95%CI	P	OR	95%CI	P
Unadjusted	1	0.912	0.690–1.207	0.521	0.782	0.590–1.035	0.086	**0.702**	**0.530–0.930**	**0.014**
Age-adjusted	1	1.036	0.775–1.385	0.811	0.975	0.728–1.307	0.866	1.018	0.752–1.377	0.909
Multi-adjusted	1	1.024	0.765–1.370	0.874	0.952	0.709–1.280	0.747	0.988	0.723–1.350	0.940

^*^The levels of osteocalcin is divided into quartile (Q1 < levels of 25%, 25% ≤ Q2 ≤ 50%, 50% < Q3 ≤ 75%, Q4 > 75%).

^*^Multi-adjusted: adjust for age, smoking status, alcoholic drinking, BMI, WHR.

^*^ED = erectile dysfunction; BMI = Body Mass Index; WHR = waist hip rate.

### OC is significantly related to moderate–severe ED in middle-aged (40–49 years) men

In the regression analyses mentioned above, the significant association between OC and ED risk disappeared after adjusting for age. Correlation analyses were subsequently performed, and age was negatively associated with the OC level (r = −0.336, *P* < 0.001) and IIEF score (r = −0.188, *P* < 0.001). Therefore age may influence the relationship between OC and ED. Considering this, a subgroup analysis of age was conducted. Previous work had highlighted that 40 years of age may be the joint point for the increasing morbidity of ED^13^. Therefore the participants were divided into four age groups: <40, 40–49, 50–59, and ≥60 years. No obvious associations were discovered in the <40, 50–59, or ≥60 groups; however, the results suggested that OC may be associated with ED in the 40–49 age group, even in the multi-adjusted model (IIEF-5: β = 2.480, 95% CI = 0.995–3.965, *P* = 0.001; ED: OR = 0.368, 95% CI = 0.168–0.807, *P* = 0.013). In addition, significant correlations were mainly present in the moderate (multi-adjusted: OR = 0.255, 95% CI = 0.067–0.965, *P* = 0.044) and severe (unadjusted: OR = 0.061, 95% CI = 0.009–0.394, *P* = 0.003; age-adjusted: OR = 0.060, 95% CI = 0.009–0.397, *P* = 0.003; multi-adjusted: OR = 0.065, 95% CI = 0.009–0.440, *P* = 0.005) ED groups (Table 5).

**Table 5:** Association between ED and osteocalcin level in four ages groups (<40, 40–49, 50–59 and ≥60 years).

		No. ED	No. Non-ED	Unadjusted			Age-adjusted			Multi-adjusted		
				BETA/OR	95%CI	P	BETA/OR	95%CI	P	BETA/OR	95%CI	P
Ages <40												
IIEF-5		444	635	**−0.903**	**−1.690, −0.117**	**0.024**	−0.520	−1.368, 0.328	0.229	−0.467	−1.346, 0.411	0.297
ED				1.048	0.709–1.548	0.814	1.142	0.748–1.742	0.538	1.097	0.708–1.698	0.679
	None			1	1	1	1	1	1	1	1	1
	Mild			0.912	0.592–1.404	0.675	1.122	0.704–1.788	0.628	1.072	0.662–1.736	0.778
	Moderate			0.740	0.328–1.668	0.468	0.739	0.309–1.764	0.495	0.696	0.284–1.709	0.430
	Severe			**4.846**	**1.864–12.600**	**0.001**	2.003	0.690–5.810	0.201	2.037	0.669–6.204	0.211
40–49		189	154									
IIEF-5				**2.412**	**0.952, 3.873**	**0.001**	**2.385**	**0.930, 3.840**	**0.001**	**2.480**	**0.995, 3.965**	**0.001**
ED				**0.363**	**0.167–0.785**	**0.010**	**0.366**	**0.169–0.791**	**0.011**	**0.368**	**0.168–0.807**	**0.013**
	None			1	1	1	1	1	1	1	1	1
	Mild			0.460	0.199–1.061	0.069	0.463	0.201–1.068	0.071	0.488	0.209–1.143	0.099
	Moderate			0.308	0.085–1.114	0.073	0.311	0.086–1.124	0.075	**0.255**	**0.067–0.965**	**0.044**
	Severe			**0.061**	**0.009–0.394**	**0.003**	**0.060**	**0.009–0.397**	**0.003**	**0.065**	**0.009–0.440**	**0.005**
50–59		74	26									
IIEF-5				0.418	−2.815, 3.651	0.798	0.350	−2.905, 3.604	0.832	0.929	−2.509, 4.366	0.593
ED				0.708	0.135–3.720	0.684	0.700	0.132–3.705	0.675	0.623	0.106–3.655	0.600
	None			1	1	1	1	1	1	1	1	1
	Mild			0.551	0.087–3.473	0.526	0.557	0.089–3.480	0.531	0.497	0.071–3.480	0.482
	Moderate			1.622	0.182–14.433	0.665	1.503	0.167–13.502	0.716	1.898	0.183–19.662	0.591
	Severe			0.369	0.031–4.347	0.428	0.394	0.032–4.844	0.467	0.205	0.012–3.403	0.269
≥60		39	6									
IIEF-5				2.024	−1.726, 5.775	0.282	1.769	−1.981, 5.519	0.347	1.530	−2.366, 5.425	0.432
ED				0.631	0.071–5.583	0.631	0.551	0.057–5.352	0.608	0.237	0.016–3.570	0.298
	None			1	1	1	1	1	1	1	1	1
	Mild			0.814	0.071–9.313	0.869	0.623	0.045–8.643	0.724	0.187	0.008–4.289	0.294
	Moderate			0.792	0.069–9.108	0.851	0.741	0.058–9.478	0.818	0.276	0.013–5.714	0.405
	Severe			0.194	0.008–4.596	0.310	0.199	0.009–4.544	0.312	0.150	0.005–4.484	0.273

^*^Linear regression analysis for the IIEF-5 scores; binary regression analysis for ED; multinomial logistic regression for the severity of symptom of ED.

^*^ED is divided into four groups according to the IIEF-5 scores: None = 22–25 scores, Mild = 17–21 scores, Moderate = 12–16 scores and Severe = 5–11 scores. None ED is treated as the reference in the multinomial logistic regression.

^*^ED: erectile dysfunction; BMI: body mass index; WHR: waist hip rate; OR: odd ratio; 95%CI: 95% confidence interval.

^*^Multi-adjusted: age, BMI, WHR, smoke and drink.

**Figure 1:**
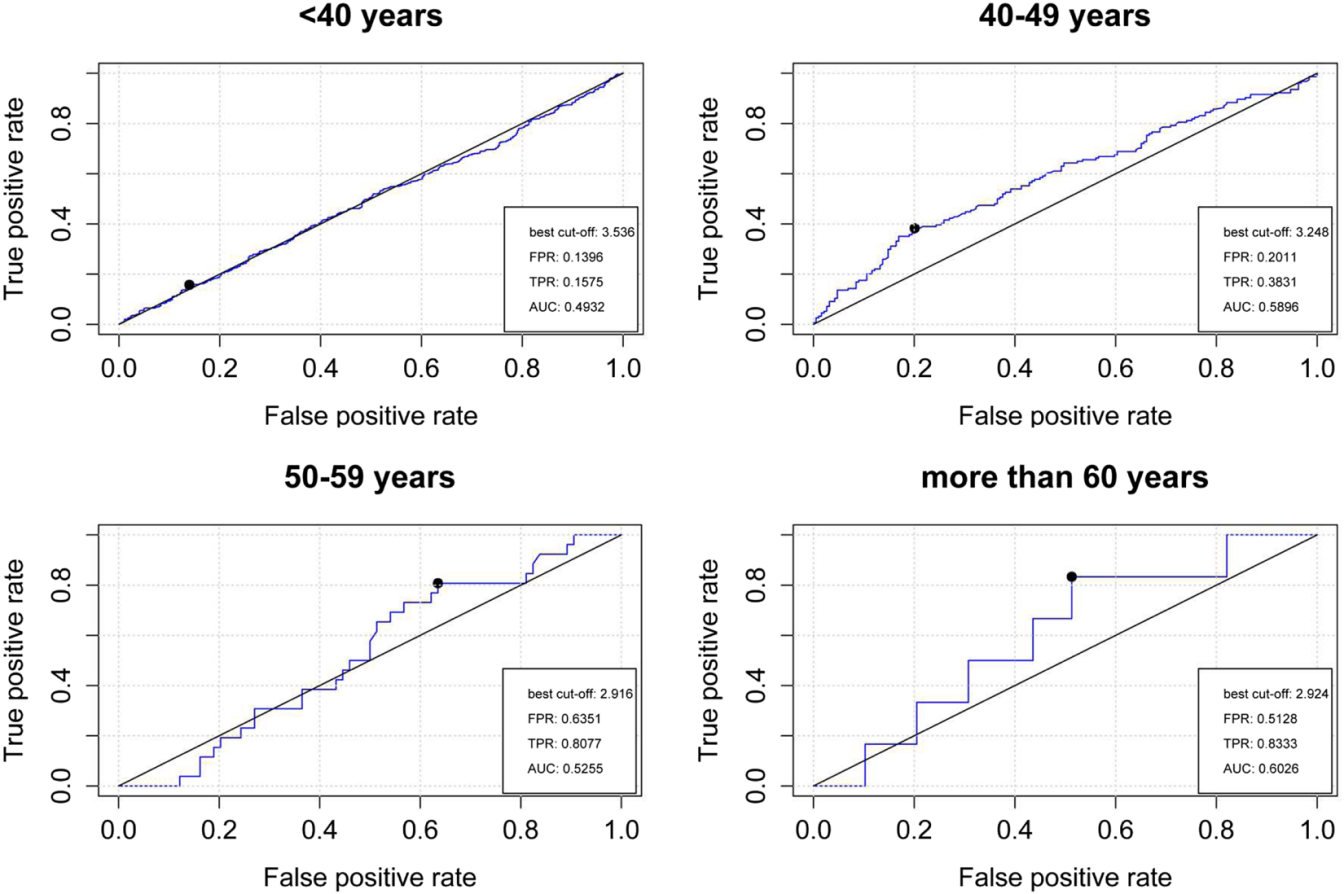
Relative operating characteristic (ROC) curve analyses of osteocalcin in predicting the ED risk in four different age groups (<40, 40–49, 50–59 and ≥60 years). The Best cut-off, area under the curve (AUC), False Positive Rate (FPR) and True Positive Rate (TPR) are shown in the figures.

In the receiver operating characteristic (ROC) analysis, OC was also shown to predict ED in the 40–49 age group with a better area under the curve (AUC = 0.5896, *P* = 0.004, best cutoff lnOC = 3.248) compared to the other age groups (Fig. 1).

### Correlation of OC and ED with higher testosterone levels

In the baseline analysis, no difference in testosterone level was detected between the ED (6.34 ± 1.85 ng/ml) and non-ED (6.23 ± 1.93 ng/ml, *P* = 0.233) groups (Table 1). Previous studies suggested that testosterone can influence the OC level and the development of ED^10^, and the level of testosterone was positively correlated with OC (r = 0.249, *P* < 0.001). To discover the influence of testosterone in the association between OC and ED, subgroup analyses were conducted after subdividing the testosterone levels into quartiles (Table 6). The 50–59 and ≥60 age groups were combined in this analysis due to the limited number of participants aged >60 years old. Once again, a significant association was only shown in the 40–49 age group. In the testosterone subgroups, the correlation between OC and ED was more prominent in those with higher testosterone levels [Q3 (unadjusted: OR = 0.077, 95% CI = 0.011–0.546, *P* = 0.010; age-adjusted: OR = 0.079, 95% CI = 0.011–0.578, *P* = 0.012; multi-adjusted: OR = 0.068, 95% CI = 0.008–0.555, *P* = 0.012) and Q4 (multi-adjusted: OR = 0.184, 95% CI = 0.034–0.985, *P* = 0.048)].

**Table 6:** The logistic regression analysis for the association between ED and osteocalcin in various groups.

		No.		Osteocalcin, ng/ml		Unadjusted		Age-adjusted		Multi-adjusted	
		ED	non-ED	ED	non-ED	OR/95%CI	P	OR/95%CI	P	OR/95%CI	P
Age < 40											
testosterone	Q1	98	168	24.27 ± 7.41	23.18 ± 7.17	1.746/0.731–4.168	0.209	1.835/0.735–4.580	0.193	1.836/0.716–4.711	0.206
	Q2	114	160	24.23 ± 7.52	25.36 ± 8.21	0.627/0.276–1.428	0.266	0.985/0.402–2.417	0.974	1.044/0.415–2.628	0.927
	Q3	108	163	26.73 ± 8.88	27.24 ± 9.29	0.794/0.362–1.742	0.565	0.808/0.343–1.901	0.625	0.788/0.319–1.948	0.606
	Q4	124	144	29.19 ± 9.04	29.42 ± 9.60	0.989/0.451–2.168	0.978	0.878/0.383–2.014	0.759	0.917/0.391–2.153	0.843
BMI	<24	272	403	28.13 ± 9.12	27.39 ± 9.26	1.351/0.830–2.199	0.226	1.531/0.896–2.616	0.119	1.477/0.850–2.568	0.166
	<28, ≥24	146	173	23.19 ± 6.59	24.65 ± 8.14	0.508/0.229–1.126	0.095	0.500/0.219–1.143	0.100	0.488/0.212–1.125	0.092
	≥28	26	59	23.44 ± 5.43	22.53 ± 6.14	2.122/0.346–13.015	0.416	2.526/0.394–16.202	0.328	1.489/0.200–11.079	0.697
WHR	≤0.9	312	448	27.53 ± 9.02	27.54 ± 9.33	1.012/0.638–1.605	0.961	1.164/0.708–1.912	0.550	1.194/0.716–1.990	0.498
	>0.9	132	187	23.17 ± 6.20	22.95 ± 6.62	1.213/0.532–2.764	0.647	1.138/0.484–2.676	0.768	1.002/0.420–2.389	0.997
Smoking	No	191	277	26.51 ± 8.17	27.10 ± 9.69	0.901/0.501–1.620	0.727	0.896/0.465–1.729	0.744	0.970/0.494–1.903	0.929
	Yes	253	358	26.02 ± 8.78	25.48 ± 8.11	1.189/0.703–2.012	0.518	1.341/0.768–2.341	0.302	1.158/0.646–2.073	0.623
Drinking	No	55	68	26.53 ± 6.87	26.97 ± 11.12	1.238/0.402–3.809	0.710	1.351/0.404–4.513	0.625	0.946/0.247–3.627	0.936
	Yes	389	567	26.19 ± 8.73	26.10 ± 8.56	1.020/0.672–1.547	0.926	1.108/0.705–1.740	0.658	1.079/0.677–1.719	0.748
MetS	No	410	575	26.57 ± 8.65	26.59 ± 8.72	1.003/0.664–1.513	0.990	1.103/0.705–1.725	0.667	1.126/0.710–1.786	0.613
	Yes	34	60	22.10 ± 5.31	22.38 ± 9.37	1.176/0.281–4.927	0.825	1.197/0.281–5.096	0.808	0.983/0.217–4.463	0.982
Age 40–49											
testosterone	Q1	44	41	21.16 ± 7.18	21.66 ± 6.75	0.787/0.204–3.031	0.727	0.778/0.201–3.007	0.716	0.955/0.224–4.077	0.951
	Q2	39	47	22.02 ± 5.26	23.61 ± 6.87	0.453/0.091–2.258	0.334	0.443/0.087–2.245	0.325	0.404/0.073–2.244	0.300
	Q3	60	26	21.43 ± 5.76	25.24 ± 6.22	**0.077/0.011–0.546**	**0.010**	**0.079/0.011–0.578**	**0.012**	**0.068/0.008–0.555**	**0.012**
	Q4	46	40	21.81 ± 4.46	25.14 ± 10.40	0.259/0.050–1.340	0.107	0.251/0.049–1.294	0.098	**0.184/0.034–0.985**	**0.048**
BMI	<24	102	82	22.02 ± 5.79	25.34 ± 8.70	**0.221/0.075–0.650**	**0.006**	**0.232/0.079–0.684**	**0.008**	**0.199/0.064–0.614**	**0.005**
	<28, ≥24	67	58	21.19 ± 5.97	22.24 ± 6.41	0.491/0.130–1.858	0.294	0.490/0.129–1.857	0.294	0.564/0.145–2.194	0.409
	≥28	20	14	20.63 ± 4.31	20.81 ± 6.60	1.449/0.134–15.630	0.760	1.545/0.137–17.420	0.725	1.669/0.141–19.744	0.684
WHR	≤0.9	93	80	22.00 ± 5.61	25.20 ± 6.71	**0.141/0.042–0.479**	**0.002**	**0.148/0.043–0.506**	**0.002**	**0.122/0.034–0.438**	**0.001**
	>0.9	96	74	21.17 ± 5.81	22.21 ± 8.75	0.756/0.272–2.096	0.590	0.746/0.269–2.071	0.574	0.890/0.312–2.544	0.828
Smoking	No	79	70	21.85 ± 5.76	24.06 ± 7.07	0.315/0.094–1.051	0.060	0.339/0.101–1.145	0.082	0.290/0.083–1.017	0.053
	Yes	110	84	21.39 ± 5.70	23.52 ± 8.53	0.408/0.149–1.115	0.080	0.398/0.145–1.087	0.072	0.424/0.153–1.177	0.100
Drinking	No	27	21	20.55 ± 4.33	23.87 ± 7.19	0.134/0.012–1.504	0.103	0.096/0.007–1.251	0.074	0.125/0.009–1.685	0.117
	Yes	162	133	21.75 ± 5.91	23.75 ± 8.01	**0.411/0.182–0.930**	**0.033**	**0.420/0.186–0.951**	**0.037**	**0.405/0.177–0.931**	**0.033**
MetS	No	157	127	22.07 ± 5.66	24.02 ± 7.01	**0.387/0.162–0.925**	**0.033**	**0.394/0.164–0.943**	**0.036**	**0.400/0.164–0.975**	**0.044**
	Yes	32	27	19.17 ± 5.47	22.56 ± 11.19	0.220/0.033–1.471	0.118	0.220/0.033–1.486	0.120	0.203/0.027–1.551	0.124
Age ≥ 50											
testosterone	Q1	28	9	19.33 ± 7.13	19.68 ± 4.40	0.569/0.058–5.597	0.629	0.549/0.056–5.354	0.606	0.539/0.057–5.053	0.588
	Q2	30	5	21.45 ± 5.89	22.74 ± 3.62	0.256/0.005–12.138	0.489	0.418/0.006–27.641	0.684	0.148/0.001–22.152	0.455
	Q3	25	12	25.50 ± 13.52	23.07 ± 5.02	1.391/0.169–11.484	0.759	1.396/0.166–11.774	0.759	0.996/0.057–17.420	0.998
	Q4	30	6	21.29 ± 5.66	21.66 ± 5.00	0.648/0.017–24.019	0.814	0.789/0.017–36.854	0.904	1.174/0.013–108.554	0.945
BMI	<24	62	15	22.22 ± 6.60	22.64 ± 5.61	0.667/0.090–4.964	0.692	0.737/0.092–5.915	0.774	0.961/0.097–9.548	0.973
	<28, ≥24	36	13	21.58 ± 11.76	22.39 ± 2.87	0.368/0.052–2.598	0.316	0.363/0.050–2.634	0.316	0.304/0.042–2.191	0.237
	≥28	15	4	20.42 ± 6.95	16.73 ± 2.26	20.079/0.051–7888.492	0.325	13.892/0.033–5856.222	0.394	NA /NA	NA
WHR	≤0.9	49	12	22.06 ± 6.53	22.43 ± 4.78	0.629/0.064–6.237	0.692	0.643/0.061–6.761	0.713	0.956/0.056–16.282	0.975
	>0.9	64	20	21.56 ± 9.89	21.42 ± 4.67	0.676/0.146–3.133	0.617	0.704/0.149–3.322	0.657	0.584/0.102–3.360	0.547
Smoking	No	66	19	22.01 ± 9.62	21.29 ± 4.51	0.918/0.167–5.027	0.921	0.896/0.162–4.948	0.899	0.891/0.152–5.206	0.898
	Yes	47	13	21.45 ± 6.90	22.55 ± 4.95	0.440/0.055–3.494	0.438	0.434/0.049–3.810	0.451	0.232/0.017–3.186	0.274
Drinking	No	28	6	24.71 ± 13.30	20.25 ± 4.91	3.924/0.163–94.649	0.400	5.666/0.126–254.731	0.372	4.168/0.080–216.801	0.479
	Yes	85	26	20.81 ± 6.10	22.16 ± 4.63	0.326/0.062–1.704	0.184	0.330/0.062–1.758	0.194	0.342/0.063–1.860	0.215
MetS	No	88	26	21.97 ± 8.94	21.91 ± 4.78	0.706/0.171–2.908	0.630	0.730/0.174–3.065	0.667	0.743/0.159–3.483	0.707
	Yes	25	6	21.11 ± 7.20	21.30 ± 4.48	0.614/0.032–11.748	0.746	0.635/0.030–13.423	0.771	0.626/0.019–20.596	0.793

^*^Testosterone levels are divided into quartile (Q1 < levels of 25%, 25% ≤Q2 ≤ 50%, 50% <Q3 ≤75%, Q4 > 75%) in different ages.

^*^BMI is categorized into three groups (normal weight <24.0 kg/m^2^, overweight 24.0–27.9 kg/m^2^, obese 28.0 kg/m^2^). Meanwhile, the waist-hip ratio (WHR) is categorized as normal weight (WHR ≤ 0.9) and obese (WHR > 0.9).

^*^Smoking, drinking and metabolic syndrome (MetS) are considered as dichotomous variable (Yes/No).

^*^ED is considered as dichotomous variable (ED/without ED). Then, binary logistic regression analyses are conducted for every subgroup, in which ED is treated as the dependent factor. And the level of osteocalcin is treated as covariate.

^*^The level of osteocalcin (ng/ml) is presented as mean ± SD in the ED and non-ED groups.

^*^ED: erectile dysfunction; BMI: body mass index; WHR: waist hip rate; OR: odd ratio; 95%CI: 95% confidence interval; MetS: metabolic syndrome.

^*^Multi-adjusted: age, BMI, WHR, smoking and drink.

**Figure 2:**
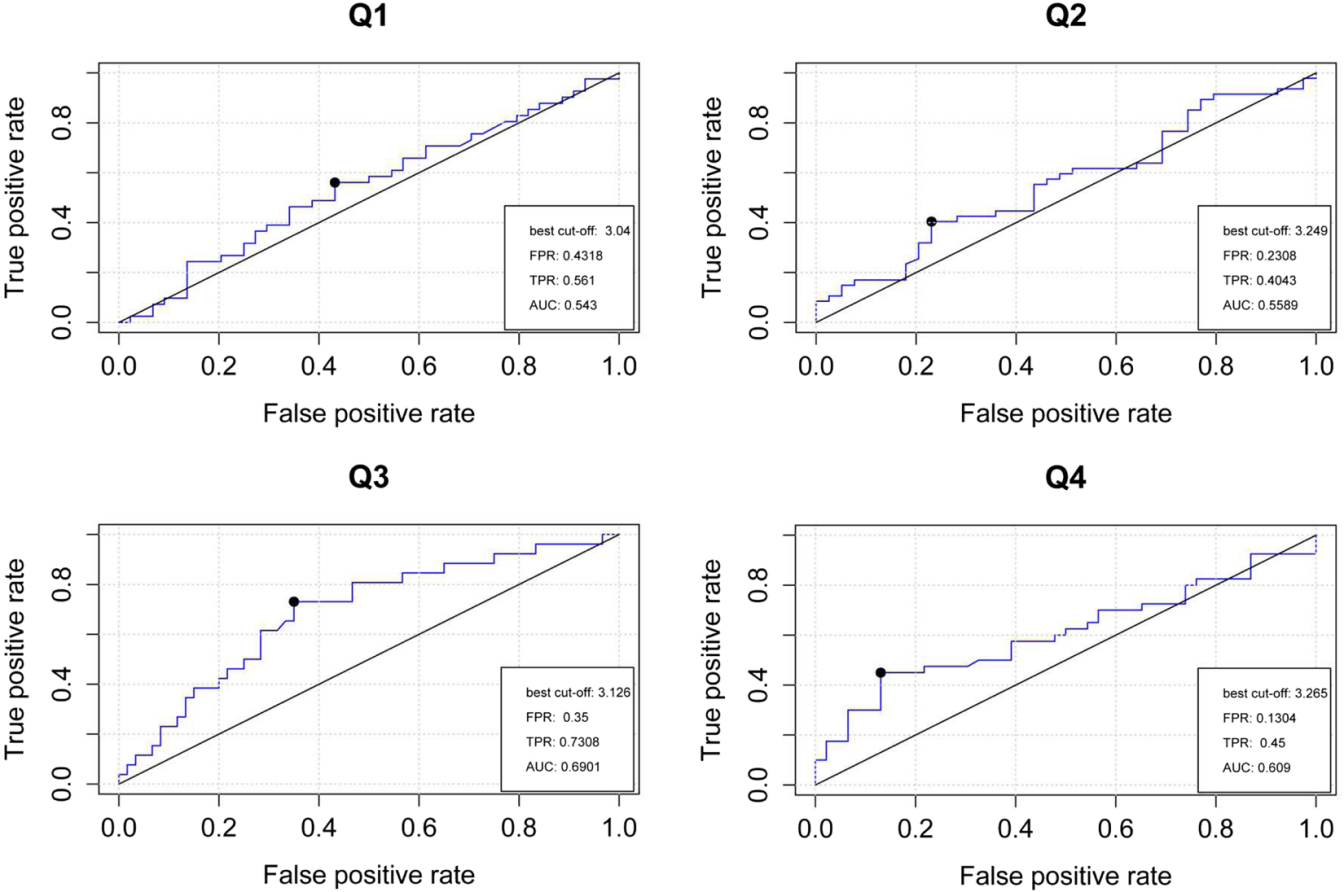
Relative operating characteristic (ROC) curve analyses of osteocalcin in predicting the ED risk in four different groups (Q1 < levels of 25%, 25% ≤ Q2 ≤ 50%, 50% < Q3 ≤ 75%, Q4 > 75%) on the basis of testosterone levels among 40–49 years. The Best cut-off, area under the curve (AUC), False Positive Rate (FPR) and True Positive Rate (TPR) are shown in the figures.

ROC analysis also indicated that OC presented an excellent predictive function for ED in those with high levels of testosterone among the 40–49 age group, with the AUC increasing to 0.6901 (*P* = 0.005). The best cut-off of lnOC was 3.126 (Fig. 2).

### OC is related to ED in the non-obese population

This study also aimed to identify the influence of obesity in the association between OC and ED. Obesity was defined using BMI (normal weight: <24.0 kg/m^2^, overweight: 24.0–27.9 kg/m^2^, obese: ≥28.0 kg/m^2^) and WHR (normal weight: WHR ≤ 0.9 and obese: WHR > 0.9). This analysis also confirmed the association between OC and the development of ED. In addition, consistent conclusions were reached that showed that the correlations were more prominent in the non-obese population [BMI < 24 kg/m^2^: (unadjusted: OR = 0.221, 95% CI = 0.075–0.650, *P* = 0.006; age-adjusted: OR = 0.232, 95% CI = 0.079–0.684, *P* = 0.008; multi-adjusted: OR = 0.199, 95% CI = 0.064–0.614, *P* = 0.005); WHR < 0.9: (unadjusted: OR = 0.141, 95% CI = 0.042–0.479, *P* = 0.002; age-adjusted: OR = 0.148, 95% CI = 0.043–0.506, *P* = 0.002; multi-adjusted: OR = 0.122, 95% CI = 0.034–0.438, *P* = 0.001)] (Table 6).

**Figure 3:**
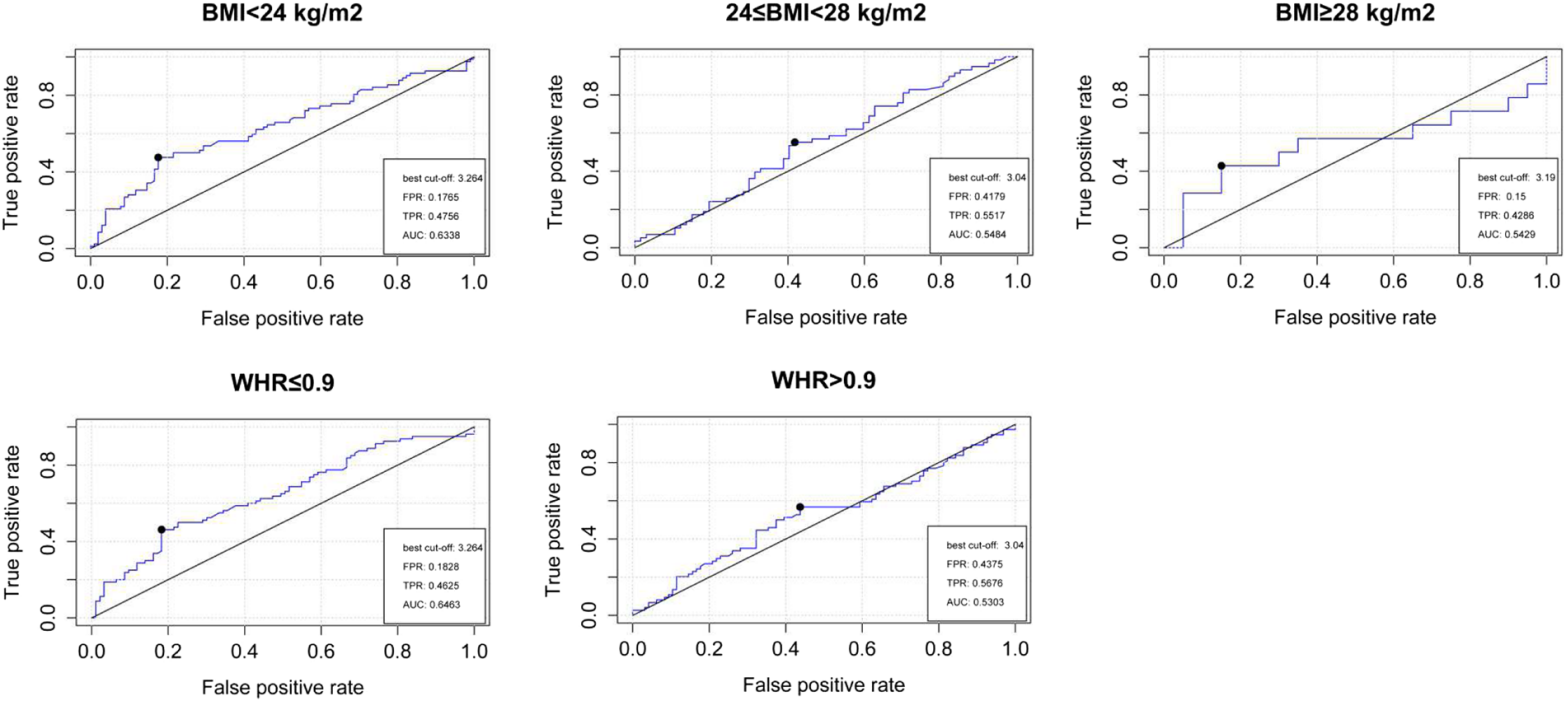
Relative operating characteristic (ROC) curve analyses of osteocalcin in predicting the ED risk for the obesity assessed by BMI and WHR. The Best cut-off, area under the curve (AUC), False Positive Rate (FPR) and True Positive Rate (TPR) are shown in the figures.

The results of the ROC analysis also indicated that better prediction occurred in the non-obese group (BMI < 24 kg/m^2^: AUC = 0.6338, *P* = 0.002; WHR < 0.9: AUC = 0.6463, *P* = 0.001), with the same best cutoff lnOC of 3.264 (Fig. 3).

### Drinking alcohol and the absence of metabolic syndrome (MetS) were associated with the potential protection of OC in ED

Among the 40–49 age group, the correlation between OC and ED was also shown in those that drank alcohol (unadjusted: OR = 0.411, 95% CI = 0.182–0.930, *P* = 0.033; age-adjusted: OR = 0.420, 95% CI = 0.186–0.951, *P* = 0.037; multi-adjusted: OR = 0.405, 95% CI = 0.177–0.931, *P* = 0.033) compared to those that did not drink. Interestingly, men without MetS seemed to be associated with the potential protection of OC (unadjusted: OR = 0.387, 95% CI = 0.162–0.925, *P* = 0.033; age-adjusted: OR = 0.394, 95% CI = 0.164–0.943, *P* = 0.036; multi-adjusted: OR = 0.400, 95% CI = 0.164–0.975, *P* = 0.044) comparing to those with MetS (Table 6).

## Discussion

The results from this study combined with previous evidence suggest that OC may be significantly associated with ED to some extent. The positive correlation between OC and ED mainly presented in middle-aged men (40–49 years) with moderate–severe ED. To explain the potential mechanisms, data on other medical factors including testosterone levels, obesity, smoking, drinking alcohol, and MetS were also collected. Further analyses revealed that the correlation between OC and ED was only significant in those individuals with higher testosterone levels who were not obese, drank alcohol, and did not have MetS.

OC is a 5.6 kDa secreted protein, containing 46–50 amino acids, that is produced primarily by osteoblasts^14^. It was first isolated from bovine and human bone by Price *et al*.^3^, and for a long time it was thought that OC manifested its effects in bone. However, OC was recently shown to play important roles in energy metabolism, fertility, and the development of CVD^6–8^. As a complex disease, ED is also associated with CVD^8^. Moreover, ED is also suggested to be a marker of subclinical CVD^15^. Previous studies also hinted that ED may be associated with OC. In 2010, one study focused on OC-positive endothelial progenitor cells in patients affected by ED and cavernous atherosclerosis, and the results suggested that the levels of OC-positive endothelial progenitor cells could be a predictive marker for subsequent coronary artery disease in ED patients^16^. Although no direct evidence has explained the association between ED and OC levels, fragmentary signals could also identify the potential correlations between them.

In 2005, experimental animal studies proposed that androgen deprivation caused stromal progenitor cells to turn into adipocytes, which was one of the most frequent causes of ED^17,18^. Meanwhile, it was also suggested that testosterone could open smooth muscle potassium channels in human penile cavernous bodies to influence the erection^19^. In 2012, data from the *FAMHES* project revealed that testosterone was associated with ED^10^. Moreover, in 2014, a meta-analysis showed that testosterone replacement therapy could lead to an 18% reduction in the risk of ED^20^. Another well-known factor, nitric oxide, was also shown to regulate the erection of the corpus cavernosum, and the expression of nitric oxide was also shown to be affected by androgens^21^. Penile nitric oxide synthase (NOS) decreased following castration but was rectified with testosterone therapy^22^. These results suggest that testosterone could protect from ED to some extent. In addition, one previous study showed that OC could favor male fertility by enhancing testosterone production^7^. In accordance with previous studies^23,24^, this study also showed that the OC level was positively associated with testosterone (r = 0.249, *P* < 0.001). Although it was reasonable to assume that OC may be associated with ED, the significance only seemed to be interesting for middle-aged (40–49 years) men. It was previously reported that testosterone decreased as rapidly as 0.4–2% per year after age 30^25^; however, the function of testosterone was special. When the level of testosterone was lower than the threshold, it was associated with a worse sexual function. Upon reaching the threshold, the effect plateaud^26^. Therefore, this study suggested that the testosterone concentration in middle-aged men was predominantly lower than the threshold. On the other hand, testosterone levels were high enough to take effect in the <40 age group. This phenomenon could be consistent with the morbidity of ED. As shown previously, the occurrence rate of ED increased in men aged >40 years^13^, therefore the protective effect of testosterone was obvious in those aged ≥40 years. Similarly, osteoporosis, metabolic abnormalities, and hormonal disorders in older men induced reduced OC and testosterone levels, which weakened the association with ED to some extent.

Another interesting result from this study was that the positive correlation between OC and ED was mainly observed in the non-obese population. An association between obesity and sex hormones was recently established, with lower levels of free and bioavailable testosterone being significantly associated with obesity^27^. In 2012, Vaidya *et al*.^28^ confirmed that testosterone was negatively associated with WHR. Yamacake *et al*.^29^ also discovered that men with a higher BMI had lower serum androgen levels. In animal experiments, testosterone levels declined in obese rats fed a high-fat diet^30^. Possible mechanisms behind this phenomenon could be obesity-associated insulin resistance, hypothalamus–pituitary–testicular axis function, or the transformation and secretion of glucocorticoids^31,32^. The inverse correlation between OC and obesity in our analysis was consistent with the results from previous studies^33,34^. Therefore the levels of OC and testosterone increased in the non-obese population, which would have enhanced the protective effect against the development of ED.

This analysis also revealed that OC was associated with ED in individuals without MetS compared to those with MetS. This phenomenon could be explained using data from other studies. In 2013, researchers discovered lower OC levels in individuals with MetS^35^. This conclusion was later confirmed in the MINOS cohort^36^. In a population study, long-term drinkers showed significantly higher levels of OC than social drinkers^37^, which was also in line with the results obtained here. These studies suggest that OC levels are higher in those without MetS and those who drink. As a regulator, this would lead to increased testosterone levels, which would subsequently protect from ED using the mechanism described above.

## Limitations

This study mainly focused on the association between OC and ED; however, some issues should not be ignored. Firstly, this study was a cross-sectional analysis and therefore it only suggests a potential protective effect of OC in the development of ED. Additional longitudinal studies are needed to confirm this conclusion. Secondly, other hormones such as luteinizing hormone and follicle-stimulating hormone may also influence the association between OC and ED. Therefore further studies should also consider these hormones more comprehensively. Thirdly, although testosterone could reflect the functional status of the testis to some extent, other factors such as semen quality were not included.

## Conclusions

The results suggest that OC may have a protective role against moderate–severe ED in middle-aged (40–49 years) men, especially those with a high level of testosterone, those that are not obese, those that drink alcohol, and those without MetS. Further studies are needed to confirm the association and the potential mechanisms.

## Methods and Materials

### Population

*FAMHES* was a population-based project that mainly focused on environmental and genetic factors as well as their interrelations. A total of 4303 men participated in a routine physical examination at the medical center in Fangchenggang First People’s Hospital between September and December 2009. Data were collected from 3593 participants using interviews. The response rate was 83.5%^13^. All participants signed written informed consent and provided a whole blood sample. The study was approved by the medical ethics committee at Guangxi Medical University. All methods were performed in accordance with their relevant guidelines and regulations.

### ED definition and sample screening

ED was defined using the International Index of Erectile Function (IIEF-5) system^38^. This system asks five questions that are mainly focused on erection confidence, erection firmness, maintenance ability, maintenance frequency, and satisfaction, with the total score ranging from 0 to 25. Each question has six answers, which score 0–5 points. Lower scores indicate a reduced sexual function and an increased severity of ED. As with the previous standard^39^, the population can be divided into four categories: no dysfunction (IIEF-5 score: 22–25), mild dysfunction (17–21), moderate dysfunction (12–16), and severe dysfunction (5–11). Moreover, an IIEF-5 score of 22 can be used to subdivide the population into those with ED (IIEF-5 ≤ 21; mild, moderate and severe dysfunction) and those without ED (IIEF-5 > 21).

Subjects were excluded from the analysis if: (i) there was no data available regarding their serum OC level (e.g., they failed to contribute a blood sample or the instrument failed to detect a value); (ii) incomplete information was available on the individual; (iii) they provided incomplete answers to the ED questions; (iv) they suffered from a disease (i.e., myocardial infarction, congestive heart failure, stroke, hyperthyroidism, rheumatoid arthritis, acquired immune deficiency syndrome, or any kind of cancer); had a history of pelvic or urinary tract trauma, surgery, inflammation or chronic hepatitis, etc.; or were taking medications (i.e., psychotropic drugs, nonsteroidal anti-inflammatory drugs, antibiotics, spironolactone, cimetidine, glucocorticoids or other steroidal drugs, etc.) which may influence the level of serum OC and the normal status. Finally, 1567 eligible samples were involved in the subsequent analyses.

### Interview and physical examination

Essential information (e.g., age, sex, smoking, drinking, etc.) was collected from eligible individuals during a face-to-face interview, and a complete physical examination (e.g., height, weight, waistline, hipline, etc.) was also performed. Smoking status and alcohol consumption were divided into “smoking/drinking” or “non-smoking/non-drinking” groups. As for the physical examination, body mass index (BMI) was calculated using body weight (measured when wearing thin clothing) and height (measured without shoes). BMI was categorized into three groups according to the Chinese standard: normal weight (<24.0 kg/m^2^), overweight (24.0–27.9 kg/m^2^), and obese (≥28.0 kg/m^2^). Meanwhile, the waist–hip ratio (WHR) was categorized as normal weight (WHR ≤ 0.9) or obese (WHR > 0.9)^40^. The waist circumference was measured at the midpoint between the inferior costal margin and the superior iliac crest on the mid-axillary line, and the hipline was defined as the maximum circumference over the buttocks.

### Metabolic syndrome (MetS) and testosterone

MetS was previously shown to be significantly associated with ED and OC^41,42^. Meanwhile, as an important hormone in men, testosterone was believed to influence the levels of OC and the development of ED^9,23^. Therefore MetS and testosterone were also included in this study.

According to the updated National Cholesterol Education Program Adult Treatment Panel III for Asian Americans^43^, MetS was defined as having three or more of the following components: (1) waist circumference ≥ 90 cm, (2) triglycerides ≥ 1.7 mmol/L, (3) HDL-C < 1.03 mmol/L, (4) blood pressure ≥ 130/85 mmHg or requiring the use of antihypertensive medications, and (5) fasting glucose ≥ 5.6 mmol/L, being previously diagnosed with type 2 diabetes mellitus, or using oral antidiabetic agents or insulin.

### Serum examination

Blood samples were transported to the Department of Clinical Laboratory at the First Affiliated Hospital of Guangxi Medical University in Nanning within 2–3 h, where they were subsequently centrifuged within 15–25 min and stored at −80 °C. The serum OC and testosterone levels were measured using an electrochemiluminescence immunoassay on a COBAS 6000 system E601 (Elecsys module) immunoassay analyzer (Roche Diagnostics, GmbH, Mannheim, Germany). Triglyceride levels, HDL-C, and serum glucose were measured enzymatically on a Dimension-RxL Chemistry Analyzer (Dade Behring, Newark, DE).

### Statistical analysis

Before the analyses, serum OC and testosterone levels were logarithmically transformed to achieve approximate Gaussian distribution. Based on their IIEF-5 scores, eligible samples were divided into those with ED (IIEF-5 ≤ 21) and those without ED (IIEF-5 > 21). Student’s t-tests and X^2^ tests were applied where appropriate. Following the baseline analysis, linear and binary regression analyses were applied to assess the association between OC and ED risk after adjusting for three different models of covariates: unadjusted, age-adjusted, and multivariate-adjusted models. In the multivariate-adjusted model, the covariates were age, smoking status, alcohol consumption, BMI, and WHR. To further detect a potential association, ED was subdivided into four groups: no dysfunction, mild dysfunction, moderate dysfunction, and severe dysfunction, and multinomial logistic regression analysis was conducted to evaluate the function of OC in the order of severity of ED. The OC level was subsequently divided into four groups (Q1 < 25%, 25% ≤ Q2 ≤ 50%, 50% < Q3 ≤ 75%, Q4 > 75%) and the lowest OC group was used as the reference. Previous studies suggested that 40 years may be the point at which the morbidity of ED increased^12^, therefore the participants were divided into four age groups: <40, 40–49, 50–59, and ≥60 years. The effect of age on the association between OC and ED risk was discussed using linear, binary, and multinomial logistic regression analyses. Besides age, testosterone, obesity, and MetS were also known to affect ED and OC to some extent^9,23,41,42^. Therefore subgroup analyses were also conducted for these influencing factors. Finally, ROC analysis was applied to assess the effects of OC in predicting the ED risk. All analyses were performed using SPSS version 16.0 software (SPSS Inc., Chicago, IL, USA) and R software. All statistical tests were two-tailed.
